# Speech Perception in Noise Predicts Oral Narrative Comprehension in Children With Developmental Language Disorder

**DOI:** 10.3389/fpsyg.2021.735026

**Published:** 2021-10-21

**Authors:** Beula M. Magimairaj, Naveen K. Nagaraj, Craig A. Champlin, Linda K. Thibodeau, Diane F. Loeb, Ronald B. Gillam

**Affiliations:** ^1^Communicative Disorders and Deaf Education, Emma Eccles Jones Early Childhood Education and Research Center, Utah State University, Logan, UT, United States; ^2^Speech, Language, and Hearing Sciences, The University of Texas at Austin, Austin, TX, United States; ^3^Callier Center for Communication Disorders, The University of Texas at Dallas, Dallas, TX, United States; ^4^Communication Sciences and Disorders, Baylor University, Waco, TX, United States

**Keywords:** auditory processing, narrative language comprehension, children, speech perception in noise, psychoacoustics, developmental language disorder

## Abstract

We examined the relative contribution of auditory processing abilities (tone perception and speech perception in noise) after controlling for short-term memory capacity and vocabulary, to narrative language comprehension in children with developmental language disorder. Two hundred and sixteen children with developmental language disorder, ages 6 to 9 years (*Mean* = 7; 6), were administered multiple measures. The dependent variable was children's score on the narrative comprehension scale of the *Test of Narrative Language*. Predictors were auditory processing abilities, phonological short-term memory capacity, and language (vocabulary) factors, with age, speech perception in quiet, and non-verbal IQ as covariates. Results showed that narrative comprehension was positively correlated with the majority of the predictors. Regression analysis suggested that speech perception in noise contributed uniquely to narrative comprehension in children with developmental language disorder, over and above all other predictors; however, tone perception tasks failed to explain unique variance. The relative importance of speech perception in noise over tone-perception measures for language comprehension reinforces the need for the assessment and management of listening in noise deficits and makes a compelling case for the functional implications of complex listening situations for children with developmental language disorder.

## Introduction

Epidemiological prevalence data from monolingual English-speaking kindergartners in the upper mid-western United States indicate that 7.4% of school-age children (95% confidence interval; 6.3–8.5%) have significant difficulty in language learning and functioning despite normal-range hearing thresholds, non-verbal intelligence, and motor abilities (Tomblin et al., 1997). A variety of terms have been used to describe such children including language impairment, language-learning disability, specific language impairment, language-learning impairment, and the recently recommended term, developmental language disorder (DLD), (Bishop et al., 2016, 2017). Language-based deficits in children negatively influence social, academic, and vocational outcomes (Stothard et al., 1998; Snowling et al., 2000). Substantial evidence supports the comorbidity of language, cognitive, and auditory processing deficits in children with DLD (Bishop, 2009; Dawes and Bishop, 2009; Ferguson et al., 2011; Tomlin et al., 2015; Moore et al., 2018; Gillam et al., 2019; Sharma et al., 2019). Clinical profiles of children diagnosed as having DLD or auditory processing disorder (APD) show remarkable overlap (Ferguson et al., 2011; Miller and Wagstaff, 2011) and are heterogeneous due to the dynamic nature of development (Pennington, 2006). Therefore, DLD is not an isolated condition given its multifactorial etiology and overlap with other neurodevelopmental disorders (Bishop, 2017).

For children with APD, a multi-disciplinary management approach is recommended (British Society of Audiology, 2018). To quantify auditory processing deficits, audiologists use a battery of audiological tests and parent/teacher questionnaires of the child's listening behavior. Clinical tests for APD are designed to assess auditory processes such as localization of sound, discrimination and pattern recognition, temporal processing, closure, binaural integration and separation (ASHA, 2005). Large-scale studies have shown that there are no strong associations between auditory processing test results and listening complaints (Moore et al., 2010; Ahmmed et al., 2014; Tomlin et al., 2015). However, more robust associations between listening concerns and language impairment have been consistently observed (Sharma et al., 2009; Moore, 2012; Snowling et al., 2018; Magimairaj et al., 2020). Thus, although auditory processing deficits are not sufficient or necessary to cause DLD, they can be associated with poor language outcomes (Bishop et al., 1999; Dawes and Bishop, 2009; Snowling et al., 2018). Furthermore, children with language impairment may perform poorly on tests of auditory processing that involve linguistic stimuli. Accordingly, in this study, the aim was to examine the contribution of auditory processing abilities to narrative language comprehension, after accounting for cognition and language ability, in a large sample of school-age children with DLD.

### Auditory Perception in Children With DLD

Psychoacoustic studies have contributed significantly to our understanding of how speech is encoded in children with DLD. Whereas, several studies in children with DLD have focused on bottom-up processing (Merzenich et al., 1996; Tallal et al., 1996), others have employed broader approaches that integrated both bottom-up and top-down processing (Montgomery and Leonard, 1998; Bishop et al., 1999; Coady et al., 2007). An example of a well-known bottom-up processing theory is that DLD results from an auditory temporal processing deficit (Merzenich et al., 1996; Tallal, 2000). Specifically, the theory holds that children with DLD have unusual difficulty processing brief or rapidly changing auditory signals, which leads to problems forming well-specified mental representations of auditory stimuli. For example, Tallal and Piercy (1975) showed that children with language impairments had unusual difficulty identifying and discriminating the serial order of brief syllable sequences but performed well on stimuli with longer formant transitions. In the same vein, Wright et al. (1997) reported that children with language impairments needed louder signals than typically developing children to detect tones in masking conditions. However, these findings could have resulted from more general cognitive impairments affecting attention, perception, and memory (Marler et al., 2002; Moore, 2012). For example, Helzer et al. (1996) found that attention mechanisms differed between children with DLD and typically developing children but not temporal processing ability. In addition, children with DLD have difficulty coordinating multiple information processing resources, which interferes with memory for serial position not just temporal order (Gillam et al., 1995; Hoffman and Gillam, 2004).

The temporal processing deficit hypothesis has been extended beyond DLD to explain a broader range of deficits observed in neurodevelopmental disorders such as dyslexia and ADD/ADHD (Habib, 2021). Advances in brain imaging studies have suggested other potential mechanisms for temporal processing deficits, which are supported by the observed comorbidities in neurodevelopmental disorders. For example, functional disconnectivity between attentional networks and language areas (Horowitz-Kraus et al., 2015; Habib, 2021) or motor coordination regions and language areas (Feng et al., 2017) has been demonstrated. Second, deficits in a range of temporal processing abilities are also associated with dyschronia (Casini et al., 2018) in which children have supra-modal difficulty with temporal representations in general, such as vocabulary for temporal terms, concept of duration, chronology, or time sense (Habib, 2021). Finally, based on the temporal sampling theory of dyslexia, the synchrony between speech events and physiological activity in the brain is impaired in individuals with dyslexia (Goswami, 2011; Goswami et al., 2016). Longitudinal research has also demonstrated that phonological awareness ability is significantly predicted by auditory temporal processing abilities in children with and without dyslexia (Goswami et al., 2021).

Researchers have demonstrated auditory-specific deficits using various behavioral and electrophysiological indices in children with and without DLD (McArthur and Bishop, 2004; Bishop and McArthur, 2005; Hill et al., 2005; Bishop et al., 2010). These include but are not limited to: Deficits in auditory frequency discrimination, age-inappropriate N1-P2-N2 complex, and reduced amplitude of late discriminative negativity. Other studies in children with learning or reading problems have added to this literature (Kraus et al., 1996; Cunningham et al., 2000; Kraus, 2001; Liasis et al., 2003; Banai et al., 2005; Kraus and Nicole, 2005; Dawes and Bishop, 2010; Hornickel and Kraus, 2013; Snowling et al., 2018). A longitudinal study by Snowling et al. (2018) found that an auditory processing deficit, as measured using a Frequency Discrimination task, was prevalent in children with DLD (*N* = 64). Still, it was not predictive of later language or reading problems. Deficits in auditory processing skills such as frequency discrimination and backward masking exist only in a subgroup of children with language impairment (McArthur and Bishop, 2004; Bishop and McArthur, 2005; Mengler et al., 2005). Electrophysiological studies assessing the auditory processing of speech have shown abnormal encoding of brief speech sounds and unstable frequency-following responses in children with reading impairment (Banai et al., 2009; Hornickel and Kraus, 2013), APD, and DLD (Basu et al., 2010; Rocha-Muniz et al., 2012). We do not elaborate on the electrophysiological studies here as it is beyond the scope of this study. Overall, studies show that a subgroup of children with DLD has auditory perceptual deficits.

The idea that auditory perceptual deficits are necessary and sufficient to cause DLD has been challenged because the gamut of language impairments seen in children with DLD cannot be explained exclusively by auditory processing deficits (Dawes and Bishop, 2009; Snowling et al., 2018). In addition, other theoretical accounts of DLD in children are more predictive of the wide range of cognitive and linguistic impairments these children present (e.g., Rice et al., 1995; Leonard et al., 1997; Ullman and Pierpoint, 2005; Montgomery et al., 2018). For example, according to the Surface Account proposed by Leonard et al. (1997), phonetic segments such as grammatical morphemes are acoustically less salient, making them vulnerable to misinterpretation and omission, especially under conditions of high cognitive-linguistic load. The combination of poor phonological representations (Gillam et al., 1995; Evans et al., 2002), fast or distorted speech, and task demands that exceed capacity limits (Hoffman and Gillam, 2004) may underlie speech perception patterns in children with DLD (Coady et al., 2007). Therefore, due to an inefficient language processing system, children with DLD need repeated exposures to learn grammatical morphemes and their functions (Montgomery and Leonard, 1998, 2006; Montgomery, 2006).

Clearly, not all children with DLD have shown auditory perceptual deficits (Helzer et al., 1996; Bishop et al., 1999; Nittrouer, 1999; Rosen, 1999), and such deficits are not always associated with or confined to rapid auditory stimuli (McArthur and Bishop, 2004; Ziegler et al., 2005; Bishop et al., 2010). In addition, experimental evidence does not support the classic auditory temporal processing theory proposed by Tallal et al. (McArthur and Bishop, 2001). Based on two small-sample studies in children with DLD (*N* = 7 and 22, respectively), Merzenich et al. (1996) and Tallal et al. (1996) reported that using acoustically modified speech in language intervention resulted in significant gains in temporal processing, speech discrimination, and grammar comprehension. However, a series of large-scale RCTs have failed to replicate the initial results, showing that interventions explicitly designed to improve auditory temporal processing yield no more gains than traditional language interventions (Pokorni et al., 2004; Cohen et al., 2005; Gillam et al., 2008; McArthur et al., 2008; Fey et al., 2011). Nevertheless, temporal processing deficits should not be overlooked only based on intervention results using acoustically modified speech because there are other potential mechanisms that can cause temporal processing problems (Habib, 2021).

### Speech Perception in Noise in Children With DLD and Comorbid Developmental Disorders

Children with DLD demonstrate poor speech perception in noise (SPiN), with particular difficulty on perception of voicing contrasts. Ziegler et al. (2005) had children with DLD and age-and language-matched controls complete vowel-consonant-vowel (VCV) identification tasks in silence and noise (fluctuating, stationary, and masking release) conditions. There were 10 children in each group. Children with DLD showed substantial perceptual deficits in the noise conditions relative to controls but subtle deficits in silence. Because the deficit in children with DLD was most significant for voicing and not for low-level temporal and spectral resolution, it was suggested that the perceptual deficits were primarily related to the feature extraction stage where acoustic information is mapped to phonetic features for speech recognition rather than the encoding of acoustic stimulus properties such as envelope and fine-structure cues.

To explain how individual differences in cognitive and linguistic abilities relate to SPiN, Torkildsen et al. (2019) examined these relationships in typically developing children (*N* = 58), children with hearing impairment (*N* = 101), and children with DLD (*N* = 16) between the ages of 5 and 12 years. SPiN significantly predicted performance on the language tasks in children with DLD and children with hearing impairment, but not in children who were developing typically. In addition, memory span and IQ did not predict variance in SPiN after controlling language ability and speech perception in quiet. The authors suggested that listening in noise placed greater demands on the language system and weak or unstable lexical/phonological representations in children with DLD may have been inadequate to support the activation of target lexical items. Alternatively, the poor quality of language input that children with DLD receive due to their auditory processing deficits, may affect their ability to learn new words. The differential relationships observed in typically developing children and children with DLD motivates further examination of the factors that affect SPiN ability in children (Magimairaj et al., 2018, 2020; Torkildsen et al., 2019). In a group comparison of 26 children with and 26 children without listening difficulties, Magimairaj et al. (2020) found that scores of children with listening concerns were significantly lower in speech perception in noise, short-term memory capacity, sentence recall, inferencing, and word retrieval speed and accuracy.

Some researchers have suggested that working memory (WM) capacity plays a vital role in SPiN in typically developing children (Sullivan et al., 2015; McCreery et al., 2017, 2020), whereas others have reported no correlation (Magimairaj et al., 2018) or weak correlations (Caldwell and Nittrouer, 2013; Nittrouer et al., 2013; Torkildsen et al., 2019) between SPiN and WM capacity. However, several studies have shown that speech perception, as measured by simple word/sentence recognition tasks in noise, is not associated with measures of language ability, especially in typically developing children (Eisenberg et al., 2000; Lewis et al., 2010; Nittrouer et al., 2013; Magimairaj et al., 2018; Torkildsen et al., 2019). Other researchers have reported a positive relationship between SPiN and vocabulary (Vance et al., 2009; Vance and Martindale, 2012; Klein et al., 2017).

Several investigators have examined the relationships among SPiN, language, and cognitive abilities in the context of children with APD (Moore et al., 2010; Rosen et al., 2010; Ferguson et al., 2011; Ahmmed et al., 2014; Tomlin et al., 2015; de Wit et al., 2018). It is noteworthy that in many of these studies, children diagnosed or suspected to have APD show remarkable overlap in cognitive profiles with children diagnosed with DLD (Ferguson et al., 2011; Miller and Wagstaff, 2011).

We know of only one study of auditory perception conducted using a moderately large sample size of 64 children with DLD (Snowling et al., 2018). Large-scale studies on the contribution of auditory perception or SPiN *relative* to cognitive-linguistic factors in children with DLD, are absent. Even though studies have documented auditory perceptual deficits in children with DLD/learning disorders, the relative contribution of these deficits to language outcomes is still not empirically well-established. This is partly because there are no large-sample studies of auditory processing in children diagnosed with DLD. In addition, separating auditory perceptual deficits from global cognitive or language deficits is not feasible when using behavioral measures and employing a small-sample extreme-group design. Due to the co-occurrence of attention/WM deficits, children with DLD are more likely to perform poorly on behavioral auditory processing tasks that demand significant attention. Previous studies comparing auditory processing in small groups of children with and without DLD have helped demonstrate the association between auditory processing and DLD, but do not establish the nature of this relationship. Finally, limited evidence suggests that in typically developing children, language ability and SPiN ability are not strongly associated, but this relationship may be significant in children with DLD (Magimairaj et al., 2018; Torkildsen et al., 2019).

### The Current Study

The main aim in this study was to assess the relative contribution of auditory processing over and above cognitive-linguistic factors to narrative comprehension in a large sample of children with DLD. The co-morbidity of language, cognitive, and auditory processing deficits in children with DLD motivated this study. Specifically, we were interested in examining children's performance on narrative (spoken) language comprehension, with age, phonological short-term memory capacity (STM), vocabulary, and three auditory processing abilities as predictors (i.e., temporal processing, frequency discrimination, and SPiN). Auditory processing measures included three measures of tone-perception (temporal processing and frequency discrimination) and three SPiN measures. The temporal processing measures were patterned after previous studies involving masking tasks and children with language impairment (e.g., Wright et al., 1997; Marler et al., 2001). The signal duration was purposefully brief to enable discrete temporal sampling. The frequency discrimination task was designed to approximate the second-formant transitions of stop consonants, which are perceptible to young children. The SPiN measures came directly from the Hearing in Noise Test for Children (Nilsson et al., 1996). Comprehension of narrative language is an essential component of academic success in elementary school-age children because children rely on it to comprehend educational information and stories (Gillam and Gillam, 2016). Extensive evidence suggests that children with DLD may have impaired temporal processing and frequency discrimination ability and deficits in SPiN (Snowling et al., 2018; Habib, 2021). In addition, children with DLD often have significant limitations in vocabulary, comprehension and use of story structure and grammar elements, causal frameworks, and the ability to integrate novel information with existing conceptual knowledge (Gillam et al., 2009; Colozzo et al., 2011). Therefore, a deficit in auditory perceptual and SPiN ability may be linked to poor language skills in children with DLD.

The primary research question was: What is the relative contribution of (predictors) auditory processing over and above phonological STM capacity and vocabulary, to the comprehension of spoken narratives (dependent variable), in children with DLD? Based on Moore et al. (2010) and Snowling et al. (2018) we predicted that tone-perception tasks would not contribute unique variance to narrative comprehension. However, we also predicted that SPiN would significantly contribute uniquely to narrative comprehension in children with DLD (Torkildsen et al., 2019; Magimairaj et al., 2020).

## Materials and Methods

### Participants

Two hundred and sixteen children with DLD participated in the study. The children were recruited from nine school districts, with 96 children from Northeast Kansas, 92 from Central Texas, and 28 from North Texas. The investigators met with school personnel to explain the purpose of the investigation and the inclusion criteria. Teachers sent study brochures to the parents of children judged to be in the bottom quartile of their classes. Research coordinators at each site met with parents interested in having their children participate in the study and scheduled those children for qualification testing if they met intake criteria based on their developmental history.

Consistent with the EpiSLI model (Tomblin et al., 1997), children were enrolled in the study if they obtained standard scores at or below 81 on two or more clusters of the *Test of Language Development: Primary:3rd edition* (*TOLD:P-3*; Newcomer and Hammill, 1997), together with a standard score between 75 and 125 (+/−1.66 SD) on the Matrices subtest of the *Kaufman Brief Intelligence Test* (*K-BIT*; Kaufman and Kaufman, 1990). In addition, children were excluded if parents reported three or more episodes of otitis media in the previous 12-month period (to minimize the effects of a transient hearing loss); a history of focal brain lesions, traumatic brain injury, cerebral palsy, seizure disorders; or severely impaired reciprocal social interaction or restricted activities listed in the DSM-IV criteria for autism spectrum disorders. Hearing and vision status was confirmed through a review of school records. Project staff screened children who had no record of screening or had results that were more than 1 year old. Hearing screenings were administered at 20 dB HL at the frequencies of 1, 2, and 4 kHz in both ears (ASHA, 1997). A vision screening was completed using Lea Symbols vision screening materials to ensure adequate vision in at least one eye with or without corrective lenses. Testing was performed in a quiet room at the children's school.

Participant demographics and qualification scores are presented in Table 1. There were more males (136) than females (80) and the *Mean Age* was 90.44 months (*SD* = 9.62). The Mean Spoken Language Quotient on the *TOLD-P3* was 73.76 (*SD* = 8.62) suggesting language abilities that were 1.2 *SD* or more below the mean. The Mean standard score on the Matrices subtest of the *K-BIT* was 96.1 (*SD* = 9.04) which indicated normal-range non-verbal intellectual abilities. Based on parent report, none of the participating children had three or more ear infections in any 12-month period.

**Table 1:** Baseline characteristics of the children who participated in the study.

	***N*** **= 216** **Mean (SD)**
Age (months)	90.44 (9.62)
*K-Bit* matrices	96.1 (9.04)
*TOLD-P:3*	73.76 (8.62)
Gender	Total (percentage)
male	136 (63%)
Female	80 (37%)
Race/ethnicity	
White (not hispanic)	100 (46%)
Black/African-American	63 (29%)
White/Latino (hispanic)	32 (15%)
Other	21 (10%)
Parental education	
Completed college	81 (37.5%)
Attended college	94 (43.5%)
Did not attend college	41 (19%)

*KBIT, Kaufman Brief Intelligence Test; TOLD: P-3 value is the Spoken Language Quotient on the Test of Language Development-Primary*.

### Dependent Measure

#### Test of Narrative Language

The *TNL* (Gillam and Pearson, 2004) was used to assess children's ability to comprehend and tell stories. Children listened to three stories told by the examiner and answered literal and inferential comprehension questions about them. After the first story, children were asked to retell it. After the second and third stories, children were shown a picture and were asked to create a story that related to the picture. Narrative comprehension abilities were represented by the total number of correct responses to questions about the three comprehension stories.

### Predictor Measures

#### Cognitive Measures

The *Non-word Repetition subtest of the Comprehensive Test of Phonological Processing*, (Wagner et al., 1999) was used to assess phonological short-term memory capacity. This subtest consisted of 18 non-word ranging in length from one to six syllables. Children were asked to repeat each word. The non-word contained no consonant clusters and followed English language phonotactics. The outcome was the number of words that were repeated accurately.

#### Vocabulary Measures

*The Picture Vocabulary subtest of the TOLD: P-3* (Newcomer and Hammill, 1997) was used to assess children's ability to understand meanings associated with spoken words. Children were presented with a spoken English word and asked to show the picture that matched the word from a choice of four pictures. The outcome was the total accuracy raw score.

*The Relational Vocabulary subtest of the TOLD: P-3* (Newcomer and Hammill, 1997) was used to assess the ability to understand word associations and express their relationships. Children were asked to describe the similarities between two items presented verbally. The outcome was the total accuracy raw score.

#### Psychoacoustic Measures

##### Tone-Perception Measures

An adaptive three-interval forced-choice (3IFC) paradigm with feedback was used for all the psychoacoustic tasks. Sounds were generated digitally and presented to both ears via headphones. The stimuli levels were measured with a Larson Davis 800B sound level meter connected to a Bruel and Kjaer NBS 9A coupler. All protocols were computerized. The tasks were administered in a quiet room with an ambient noise level of 40 dB SPL or less. Ambient noise levels in the testing rooms were measured with a Radio Shack digital sound level meter (model 33-2055). The settings were “slow” and “C-scale” for the temporal response and frequency weighting, respectively.

A training session was conducted to familiarize each child with the test procedures. Colorful pictures, one corresponding to each of the listening intervals, were displayed on the computer screen. The examiner explained that there were three intervals. The child was instructed to indicate the one that sounded different by clicking the mouse pointer on the appropriate picture. Following the spoken instructions, the examiner provided a demonstration trial via a loudspeaker. The target signal was a 1-kHz tone with a starting level of 55 dB peak SPL. The total duration was 20 ms, and the signal was gated with a 10-ms cosine-squared function. The signal was randomly presented in one of the three observation intervals. The observation intervals were 350 ms in duration and were separated by a 500-ms inter stimulus interval. After a correct response was obtained on the first trial, the signal parameter for all subsequent trials were automatically adjusted based on the child's response. The signal became easier to hear after each incorrect response and more challenging to hear after two consecutive correct responses. That is, an adaptive one-up two-down procedure was used to track 71% threshold. Children were directed to guess when they were not sure of the different one. A colorful picture appeared on the screen after each correct response, but a solid green bar appeared after each incorrect response. The child was allowed to perform five practice trials independently. The headphones were then positioned over the child's ears, and five practice trials were conducted. To ensure that the child understood the experimental tasks, similar training was undertaken for each test of the tone perception tasks. During familiarization training, the criterion for completion was 80% correct (four out of five practice trials). If the criterion was not met, the training was repeated.

During testing, the examiner sat behind the child and did not provide any assistance. When ready, the child selected one of three buttons to begin the test. Three intervals were presented, and the child chose the one s/he thought contained the signal or was different. If the response was incorrect, the first trial was repeated, systematically increasing the signal parameter, until the child responded correctly. After a correct response was obtained on the first trial, the signal parameter on all subsequent trials was adaptively adjusted based on the child's response.

A test run consisted of 60 trials. A threshold estimate was based on the average reversals for the last 10 to 12 runs. Threshold variability was based on the standard deviation as calculated from the same 10 reversals. If threshold variability was greater than twice the small step size, the run was repeated. A maximum of three runs was attempted per test. The first run to satisfy the variability criterion was taken as the estimate of threshold. The child received verbal encouragement and a tangible reward upon successful completion of each run. Short rest breaks were taken between tests. Additional aspects of each test are described next.

*Simultaneous Masking:* Simultaneous masking was used to measure the child's ability to detect a brief signal in the presence of masking noise. The target signal was a 20 ms, 1-kHz tone with a starting level of 95 dB peak SPL. The masker was a narrow-band noise that extended from 0.6 to 1.4 kHz. Because the signal duration was brief, the bandwidth was wider than typical narrow-band noise to ensure effective masking. Beyond the two cut-off frequencies, the masker level decreased at the rate of −96 dB/octave. The overall duration of the masker was 300 ms, and its envelope was shaped with a gating function that included 10-ms rise/fall times. In simultaneous masking, the onset of the masker and signal were coincident. The power spectrum level of the masker was about 42 dB/Hz (overall level = 71 dB SPL). Similar to the tone-perception measures (Tone-Perception Measures), a 3IFC procedure was used to track signal threshold. The signal and masker were randomly assigned to one of the three intervals; the other intervals contained the masker alone. The intensity of the signal varied based on the child's response. The initial large step size was 4 dB for the first three reversals, after which it was reduced to 2 dB.

*Backward Masking*: The same signal and masker were used as in simultaneous masking (*Simultaneous Masking)*. However, in this task the onset of the target signal preceded the onset of the masker by 20 ms (the duration of the signal) and the starting level of the signal was 75 dB peak SPL. The 3IFC procedure was used, as previously described. One interval contained a target signal (a 20 ms, 1-kHz tone) that was immediately followed by a narrow-band masking noise. The other two auditory intervals consisted of the narrow-band masking noise presented without a target signal. The adaptive step sizes were the same as those used for simultaneous masking.

*Frequency Discrimination*: The differential threshold for 1 kHz was measured using a frequency-sweep discrimination task. In this task, the target signal was a tone that started always at 1 kHz and glided to a higher frequency. The maximum limit for the frequency glide was 8,000 Hz. The target signal was randomly assigned to one of the three intervals; the other intervals contained a steady 1 kHz tone. Based on the child's response the glide stop frequency was varied (increased for an incorrect response and decreased for two correct responses). The initial large step size was 414 Hz for the first three reversals, after which it was reduced to 72 Hz. The frequency sweep stimuli were presented at 70 dB SPL with an overall duration of 80 ms.

##### Speech Perception in Noise Measures

Children received separate training for SPiN tasks. Children were instructed that they would hear a man reading a sentence, and the loudness of the man's voice would change during testing. Sometimes the voice would be very faint. Children were told to repeat everything they heard, and that it was okay to guess if they only heard part of the sentence. The sentences were from the *Hearing in Noise Test* (HINT-C; Nilsson et al., 1996). There were 13 lists, and each list contained 10 short (6–7 syllable) sentences. The sentences were in American English and were normed on children as young as 6 years of age. This test was administered under headphones using the commercial version of the HINT-C. The background noise used was digitally filtered to match the long-term average spectrum of the test sentences and was turned on before the sentence onset and after the sentence offset.

After providing the verbal instructions, the headphones were positioned over the child's ears. The examiner, seated at a computer console with the screen facing away from the child, presented one of the pre-recorded sentences. The child responded, repeating as much of the sentence as they understood. Next, the examiner compared the child's response to a sentence text that appeared on the screen. A response was considered correct if the child repeated all the words in the sentence. The response was also correct if the child added a word to the sentence but said all the other words correctly. Additionally, contracted, or uncontracted words were acceptable, as were specific word substitutions, which appeared on the screen in parentheses. If the response was correct, the examiner selected the “Yes” button on the screen; otherwise, the “Repeat” button was selected. Choosing “Repeat” caused the sentence to be presented again, at a level that was 4 dB higher than the first presentation. This process, selecting “Repeat” and increasing the level, recurred until the child responded correctly, after which the examiner chose the “Yes” button. Once the “Yes” button was selected, the “No” button replaced the “Repeat” button, and a second sentence was presented at a level 4 dB lower than the first presentation. This process continued, with the level decreasing after a correct response and increasing after an incorrect response, until 10 sentences were presented. Verbal feedback was provided during training, and the children were encouraged to ask questions.

The procedure for testing was identical to training, except that no feedback was given. Once the child repeated the sentence, the examiner determined the correctness of the response, the presentation level was automatically adjusted, and a second sentence was delivered. The sentence became easier to hear after each incorrect response and more difficult to hear after each correct response. The large step size was 4 dB, and the small step size was 2 dB for all the speech in noise testing.

A test run consisted of 20 sentence presentations (trials). A threshold estimate was based on the average of the last 17 sentence levels. Threshold variability was based on the standard deviation as calculated from the same 17 levels. If the threshold variability was >twice the small step size (2 dB), the run was repeated. A maximum of three runs was attempted per test. The first run to satisfy the variability criterion was taken as the estimate of threshold. The child received verbal encouragement and a tangible reward upon successful completion of each run. Short rest breaks were taken between tests.

Two spatial listening conditions were simulated: “un-shadowed” and “shadowed.” The “un-shadowed” condition was analogous to listening to speech and noise coming from a sound source directly in front of the head in the sound field. In the “un-shadowed” condition, the noise was presented at an overall level of 65 dB SPL (on the A-weighting scale) and was configured to match the average long-term spectrum of the speech sentences. In the “shadowed” condition, the speech remained in front of the head, but the noise was adjusted such that it was perceived to be coming from the left side or the right side of the head (HINT-L/HINT-R) to simulate listening in sound field to speech and noise coming from separate sources or locations. The noise-right and noise-left conditions were created by reducing the noise level in the “shadowed” ear by ~20 dB. In the HINT-R configuration of the “shadowed” condition, the noise in the right ear was 65 dB SPL, while the noise in the left ear was about 45 dB. Noise levels were reversed for the two ears in the HINT-L configuration. A composite, average score from the three HINT-C subtests (un-shadowed and shadowed) was used to represent SPiN ability.

## Results

Descriptive statistics, correlation and regression analyses were conducted to determine the relative contribution of predictors (auditory processing–tone perception, SPiN; cognition–phonological STM capacity; and language ability–vocabulary) to the outcome variable (narrative comprehension), in children with DLD.

### Descriptive Statistics and Correlation Analysis

Table 2 presents the descriptive statistics and correlations. Correlations between tone perception tasks and SPiN tasks were small but significant. All tone perception tasks were positively and significantly associated with age, non-verbal IQ, phonological STM capacity, and narrative comprehension subtests that included pictures, but not comprehension of the story narrated without any picture prompt (*auditory-only*). SPiN, vocabulary, STM, and *auditory-only* comprehension, did not correlate significantly with non-verbal IQ. The three narrative comprehension subtests correlated positively with each other and generally with most predictor measures (i.e., vocabulary, phonological STM capacity, and both categories of psychoacoustic measures). The composite score from the three narrative comprehension tasks was used as the outcome variable for regression analysis. A composite, average score from the three HINT-C subtests (un-shadowed and shadowed) represented SPiN.

**Table 2:** Means, standard deviations, and correlations with confidence intervals.

**Variable**	***N***	***M***	***SD***	**1**	**2**	**3**	**4**	**5**	**6**	**7**	**8**	**9**	**10**	**11**	**12**
1. Age	216	90.44	9.62												
2. Non- verbal IQ	215	96.10	9.06	−0.06											
				[−0.19, 0.08]											
3. Non-word Rep	216	6.99	3.04	0.25**	0.09										
				[0.12, 0.37]	[−0.04, 0.22]										
4. Narrative comp	214	19.65	6.63	0.57**	0.15*	0.43**									
				[0.47, 0.66]	[0.02, 0.28]	[0.31, 0.53]									
5. Auditory -only comp	214	6.68	2.69	0.39**	0.13	0.35**	0.81**								
				[0.27, 0.50]	[-0.00, 0.26]	[0.23, 0.46]	[0.76, 0.85]								
6. Seq pict comp	214	7.36	2.65	0.59**	0.15*	0.35**	0.85**	0.51**							
				[0.50, 0.67]	[0.01, 0.28]	[0.23, 0.46]	[0.80, 0.88]	[0.41, 0.61]							
7. Single pict comp	214	5.61	2.64	0.45**	0.10	0.37**	0.84**	0.49**	0.59**						
				[0.33, 0.55]	[−0.04, 0.23]	[0.25, 0.48]	[0.79, 0.87]	[0.38, 0.59]	[0.50, 0.67]						
8. TSN	216	83.53	7.02	−0.17*	−0.15*	−0.17*	−0.22**	−0.12	−0.26**	−0.18*					
				[−0.30, −0.04]	[−0.28, −0.01]	[−0.30, −0.04]	[−0.35, −0.09]	[−0.25, 0.01]	[−0.38, −0.13]	[−0.30, −0.04]					
9. TBN	216	64.17	18.96	−0.27**	−0.21**	−0.16*	−0.22**	−0.06	−0.24**	−0.25**	0.73**				
				[−0.39, −0.14]	[−0.33, −0.08]	[−0.29, −0.03]	[−0.34, −0.09]	[−0.19, 0.08]	[−0.36, −0.11]	[−0.37, −0.12]	[0.66, 0.79]				
10. FWD	216	3464.83	2294.25	−0.27**	−0.28**	−0.17*	−0.18**	−0.06	−0.16*	−0.21**	0.46**	0.56**			
				[−0.39, −0.14]	[−0.40, −0.15]	[−0.30, −0.04]	[−0.30, −0.04]	[−0.20, 0.07]	[−0.29, −0.03]	[−0.34, −0.08]	[0.35, 0.56]	[0.46, 0.65]			
11. HINT-C	216	−1.96	2.41	−0.38**	−0.03	−0.23**	−0.50**	−0.36**	−0.44**	−0.46**	0.19**	0.21**	0.24**		
				[−0.49, −0.26]	[−0.16, 0.10]	[−0.35, −0.10]	[−0.60, −0.40]	[−0.47, −0.24]	[−0.54, −0.32]	[−0.56, −0.34]	[0.06, 0.32]	[0.08, 0.34]	[0.11, 0.36]		
12. PV	216	13.30	4.31	0.52**	0.05	0.33**	0.56**	0.37**	0.53**	0.48**	−0.14*	−0.21**	−0.11	−0.37**	
				[0.42, 0.61]	[−0.08, 0.18]	[0.20, 0.44]	[0.46, 0.64]	[0.25, 0.48]	[0.43, 0.62]	[0.38, 0.58]	[−0.27, −0.01]	[−0.34, −0.08]	[−0.24, 0.03]	[−0.48, −0.25]	
13. RV	216	8.25	4.15	0.54**	0.06	0.24**	0.47**	0.35**	0.44**	0.39**	−0.08	−0.17*	−0.21**	−0.30**	0.38**
				[0.44, 0.63]	[−0.08, 0.19]	[0.11, 0.36]	[0.36, 0.57]	[0.22, 0.46]	[0.33, 0.55]	[0.27, 0.50]	[−0.21, 0.05]	[−0.30, −0.04]	[−0.33, −0.07]	[−0.41, −0.17]	[0.26, 0.49]

*M and SD are used to represent mean and standard deviation, respectively. Values in square brackets indicate the 95% confidence interval for each correlation. The confidence interval is a plausible range of population correlations that could have caused the sample correlation (Cumming, 2014)*.

* *indicates p < 0.05*.

** *indicates p < 0.01*.

*Narrative comp, Composite narrative comprehension score; Auditory comp, Comprehension of spoken narrative with no picture cues; Seq Pict comp, Comprehension of narrative with a sequence of five pictures; Single Pict comp, Comprehension of narrative with single scene picture; TSN, Tone perception in simultaneous masking; TBN, Tone perception in backward masking; FWD, Frequency discrimination; HINT-C, Hearing in Noise Test Composite scores; PV, Picture vocabulary; RV, Relational vocabulary*.

### Regression Analysis

Multiple linear regression analysis was conducted to predict comprehension of spoken narratives from the relative contributions of vocabulary, tone-perception ability, speech perception in quiet and in noise (Table 3). Based on the theoretical notion explained in the introduction section (*1.3*, The Current Study), stepwise regression analysis was conducted to assess the relative importance of auditory processing ability (tone-perception and SPiN) to narrative comprehension after controlling for cognitive-linguistic factors. Age, non-verbal IQ, and speech perception in quiet were entered in Step 1 to factor out their effects on narrative comprehension. Speech perception in quiet was also included in Step 1 to control for potential language-based factors in the HINT-C sentences. These factors accounted for 36% of the unique variance, indicating that older children and children with higher non-verbal IQs accounted for a significant amount of variability in narrative comprehension. Phonological STM capacity was entered in Step 2 to determine the extent to which the memory measure predicted narrative comprehension over and above age and non-verbal IQ. Phonological STM capacity contributed significantly, explaining an additional 8% of the unique variance in narrative comprehension. Vocabulary (as indexed by picture vocabulary and relational vocabulary) was entered in Step 3. Vocabulary contributed significantly by explaining an additional 6% of the unique variance in addition to the previously entered predictors in Step 1 and 2. The tone-perception tasks were entered in Step 4 and they did not explain any unique variance in narrative comprehension. In the final step, SPiN when added to the model significantly accounted for 6% unique variance in narrative comprehension after controlling for all other predictors. Results remained unchanged even when the order of entering the psychoacoustic measures was reversed. In the final model, variance inflation factors (VIF) for all predictors (except TSN and TBN) were below 2.1, which suggested no threat of multi-collinearity. TSN and TBN had a VIF of > 2.1; however, both were used to represent the same construct in Step 4. Overall, the results indicated that when controlling for age, non-verbal IQ, phonological STM capacity, vocabulary, tone-perception ability, and speech perception in quiet, children are likely to have better narrative comprehension abilities if they perform well on a measure of SPiN.

**Table 3:** Summary of hierarchical linear regression analysis for predicting narrative language comprehension ability in children with developmental language disorder, using the predictors phonological short-term memory, vocabulary, tone-perception ability, and speech perception in quiet and noise.

	**Step 1**			**Step 2**			**Step 3**			**Step 4**			**Step 5**		
**Variable**	**B**	**SE B**	**β**	**B**	**SE B**	**β**	**B**	**SE B**	**β**	**B**	**SE B**	**β**	**B**	**SE B**	**β**
Age	0.32	0.04	0.47***	0.29	0.04	0.42***	0.17	0.05	0.25***	0.18	0.05	0.26***	0.15	0.04	0.22**
Non-v-IQ	0.39	0.12	0.21**	0.33	0.11	0.18**	0.28	0.11	0.15*	0.30	0.11	0.16***	0.34	0.10	0.18**
SIQ	−0.00	0.07	−0.00	0.05	0.07	0.04	0.06	0.06	0.05	0.07	0.06	0.06	0.17	0.06	0.15
PSTM				0.64	0.12	0.30***	0.50	0.11	0.23***	0.49	0.11	0.23***	0.47	0.11	0.22***
PV							0.38	0.09	0.25***	0.36	0.09	0.24***	0.26	0.09	0.17**
RV							0.22	0.09	0.14*	0.24	0.09	0.15*	0.21	0.09	0.13*
TSN										−0.15	0.07	−0.16*	−0.15	0.06	−0.16*
TBN										0.03	0.03	0.09	0.03	0.02	0.08
FWD										0.00	0.00	0.06	0.00	0.00	0.10
SPIN													−0.81	0.15	−0.30***
*Δ*R*^2^*		0.36**			0.08**			0.06**			0.01			0.06**	
*ΔF*		38.87**			29.96**			12.34**			1.87			29.43**	

*** *p < 0.001*;

** *p < 0.01*;

* *p < 0.05. Full model with all predictors: F(10, 202) = 27.27, R^2^ = 0.55, p < 0.001. Non-v-IQ, Non-verbal IQ-Matrices subtest from the Kaufman Brief Intelligence Test; SIQ, Speech perception in Quiet; PSTM, (phonological short-term memory)–Non-word repetition; PV, Picture vocabulary; RV, Relational vocabulary; TSN, Tone perception in simultaneous masking; TBN, Tone perception in backward masking; FWD, Frequency discrimination; SPIN, Speech Perception in Noise (Hearing in noise test composite score); ΔR^2^, R^2^ change; ΔF, F change; B, Unstandardized coefficients; SE, Standard error; β, Standardized coefficients Beta*.

## Discussion

The analyses presented were conducted on data collected from a sample of 216 children with DLD. The goal of the present study was to determine the relative contribution of auditory processing skills (temporal processing, frequency discrimination, and SPiN) over and above language (vocabulary) and cognitive ability (non-verbal IQ and phonological STM) to spoken narrative comprehension ability in children with DLD. The impetus for doing so was the limited evidence in the literature that examines these multiple factors in a large sample of children diagnosed with DLD. Our main finding was that psychoacoustic tone perception tasks that evaluated auditory processing skills such as simultaneous masking, backward masking, and frequency-sweep discrimination, did not explain unique variance in narrative comprehension over and above age, non-verbal IQ, speech perception in quiet, STM capacity, and vocabulary ability. This finding was similar to Snowling et al. (2018) who reported that frequency discrimination deficits were not predictive of later language abilities in children with DLD. However, SPiN was a significant predictor of children's narrative comprehension, explaining unique variance over and above all other predictors (Tomlin et al., 2015; Torkildsen et al., 2019). The HINT-C sentence repetition task used in the current study to measure speech perception in noise is similar to the sentence repetition task that children with DLD are known to have difficulty with, mainly when complex and long sentences are used (Conti-Ramsden et al., 2001; Archibald and Joanisse, 2009). Accordingly, it is not surprising that sentence repetition tasks such as SPiN predict language comprehension. Studies have also shown that vocabulary contributes to SPiN performance in typically developing children (McCreery et al., 2017, 2020). However, it is important to note that short simple sentences used in the HINT-C test were designed to assess speech perception in young children (Soli and Wong, 2008) and not language ability. The sentences for the HINT-C were adapted from *Bamford-Kowal-Bench* (BKB) sentences. A recent study reported that speech perception in noise measured using BKB sentences was not associated with typically developing school-age children language ability (Magimairaj et al., 2018). Furthermore, we controlled for children's language ability by including vocabulary and HINT-C sentence repetition in quiet, in the regression analysis. Hence, it is unlikely that the association found in this study between SPiN and narrative comprehension in children with DLD, was due to linguistic overlap of the materials used.

In previous studies, it has been demonstrated that non-verbal IQ does not load on verbal measures, auditory attention or verbal WM but instead is closely related to domain-general attention (i.e., an executive attentional resource that is shared across all modalities), (Magimairaj and Montgomery, 2012; Magimairaj et al., 2018). Interestingly, we found that tone-perception tasks were not associated with *auditory-only* comprehension of narrative language but were significantly correlated with age, non-verbal IQ, narrative comprehension subtests that included pictures, and were weakly correlated with phonological STM capacity (Table 2). This suggested the significant role of domain-general attention in tone perception (relative to domain-specific verbal factors). In addition, the tone-perception tasks were non-verbal and thus had no or low linguistic demand. In contrast, the lack of a significant correlation between non-verbal IQ and SPiN measures indicated the opposite (significance of domain-specific verbal factors over general attention for listening in noise).

The associations of phonological STM capacity and vocabulary with spoken language comprehension ability are well-established in the literature and were not of primary interest to this study. Of interest was empirical evidence to substantiate the growing body of literature on the comorbidity of auditory processing, cognitive, and language deficits in school-age children. It has been consistently indicated that models of auditory processing need greater empirical support to better establish the complex relationships between auditory processing and children's daily language performance. Thus, a multifactorial approach to delineate the role of auditory processing abilities in functional performance is essential. This study is the first to present these relationships in one of the most extensive samples of children classified as DLD. Study results highlight that individual differences in children's listening ability in noise are significant factors that uniquely predict their spoken language comprehension. Although the clinical significance of the unique variance is not quantified here, in the context of the developmental learning period, we consider this meaningful given the nature and time window of neuroplasticity. Narrative comprehension is an important functional skill that is strongly associated with academic success in elementary school children (Gillam and Gillam, 2016).

### Implications of Listening Difficulties in Noise

Estimates suggest that around 5% of children with a parental concern of listening and communication problems have normal-range hearing sensitivity (Hind et al., 2011). Children with listening difficulties frequently have trouble understanding a teacher's or friend's speech in a noisy classroom or playground where they spend a significant portion of their day (Magimairaj et al., 2020). The consequences are lost opportunities for learning and socialization during a critical period. When listening in complex auditory environments, spatially separated distracting sounds make it difficult to focus on target speech and this can be particularly challenging for children because their cognitive and language abilities are still maturing. The current study is the first to demonstrate in a large sample of children with DLD, that SPiN uniquely contributes to spoken language comprehension, even after controlling for other candidate predictors.

Substantial evidence has shown that listening difficulty in noise exists in children with spoken language comprehension problems/DLD and attention disorders (Dawes and Bishop, 2009; Sharma et al., 2009, 2019; Moore et al., 2018; Magimairaj et al., 2020). Children are particularly vulnerable to the effects of noise on spoken communication, and this is exacerbated for children with DLD, attention deficits, and hearing loss. In a recent study, along with poor SPiN scores, children with reported listening difficulties scored lower on STM capacity, sentence recall, inferencing ability, and reduced speed and accuracy of lexical access, compared to a control group (Magimairaj et al., 2020). Furthermore, Nagaraj and Magimairaj (2020) found that lexical knowledge was crucial for auditory closure of words and sentences in children, with lexical retrieval from LTM significantly associated with the recognizing interrupted sentences.

Persistent challenges in speech communication can lead to poor phonological representation, reading and learning difficulties in children (Shield and Dockrell, 2008). Whereas, the diagnosis and intervention for APD remain controversial (Moore, 2018; Iliadou et al., 2019; Neijenhuis et al., 2019), researchers and clinicians agree that speech perception deficit in noise is one of the hallmark symptoms in children who have or are suspected to have APD (American Academy of Audiology, 2010; Cameron et al., 2014; DeBonis, 2015; British Society of Audiology, 2018). Results from this study in children with DLD present the unique relationship between children's listening ability in noise and their language comprehension ability. Doing so is crucial to validate the influence of listening difficulties in noise on children's day-to-day language learning and functioning.

The auditory system is an essential gateway for speech and language processing and the selected outcome such as narrative comprehension is integral to academic success in elementary school children. The current study results have clinical implications given parent and teacher concerns about listening difficulties in children especially in noisy listening situations, and the lack of studies that model these relationships. Furthermore, an increase in age or WM capacity does not provide an advantage to children's listening ability in noise relative to a no-noise condition (Nagaraj et al., 2020). This makes it critical to enhance speech in children's learning environments using pedagogical approaches, reducing noise in the background, and providing assistive listening devices, in addition to improving children's lexical knowledge and lexical retrieval mechanisms (Magimairaj et al., 2020; Nagaraj and Magimairaj, 2020). The use of remote microphone technology in children with language impairments has shown significant improvements in speech recognition and comprehension in noise (Schafer et al., 2014).

### Future Directions

A significant number of children have SPiN deficits and poor academic and social communication outcomes. Therefore, it is crucial to understand the potential link between listening ability in noise and language and literacy outcomes. Future studies employing a battery of tasks that assess spatial listening ability in noise and language/learning outcomes are needed to explore these relationships. Findings will have implications for assessment and intervention in children who have difficulties understanding spoken language with comorbid auditory processing deficits. It is also well-established that binaural processing (Moore et al., 1991; Hall et al., 1998) and spatial listening ability in noise (Cameron et al., 2014; Tomlin and Rance, 2014; Graydon et al., 2017) are impaired in children with a history of recurrent otitis media with effusion. Recent evidence suggests that children have persistent listening difficulty in noise even after the middle ear infection has been successfully treated and hearing sensitivity reverts to normal limits (Hunter et al., 2020).

Emerging evidence shows that listening (to speech) training in spatialized noise can improve spoken language processing in complex auditory environments (Cameron et al., 2015; Graydon et al., 2018). Replication studies of this research are much needed. Understanding the role of auditory perception in real-world listening environments is crucial for guiding interventions that target mechanisms such as spatial listening and lexical retrieval abilities to improve children's speech perception.

## Conclusions

Auditory processing skills measured with tone-perception tasks did not explain variance in children's narrative comprehension. However, SPiN ability uniquely explained a significant amount of variance in children's narrative comprehension ability over and above multiple predictors such as age, non-verbal IQ, speech perception in quiet, phonological STM capacity, vocabulary, temporal processing, and frequency discrimination ability. These findings make a compelling case for the functional implications of noisy listening situations for children with DLD. To this end, further research related to assessment and intervention for listening in noise ability is warranted.

## Data Availability Statement

The raw data supporting the conclusions of this article will be made available by the authors, without undue reservation.

## Ethics Statement

The studies involving human participants were reviewed and approved by the University of Texas at Austin, the University of Texas at Dallas, and the University of Kansas. Written informed consent to participate in this study was provided by the participants' legal guardian/next of kin.

## Author Contributions

All authors listed have made a substantial, direct and intellectual contribution to the work, and approved it for publication.

## Funding

This research was funded by NIH grant U01 DC04560 awarded to RG, DL, and Sandy Friel-Patti. It also was supported by resources from NIH Grant P30 HD02528, at the Kansas Mental Retardation and Developmental Disabilities Research Center; BNCD, P30 DC005803 at the University of Kansas; and the Lillywhite Endowment at Utah State University.

## Conflict of Interest

RG receives royalties from the sale of the Test of Narrative Language, which was administered to participants. The remaining authors declare that the research was conducted in the absence of any commercial or financial relationships that could be construed as a potential conflict of interest.

## Publisher's Note

All claims expressed in this article are solely those of the authors and do not necessarily represent those of their affiliated organizations, or those of the publisher, the editors and the reviewers. Any product that may be evaluated in this article, or claim that may be made by its manufacturer, is not guaranteed or endorsed by the publisher.
